# Morbidity patterns in general practice settings of the province of Sousse, Tunisia

**DOI:** 10.4314/pamj.v3i1.52450

**Published:** 2009-11-02

**Authors:** Ridha Gataa, Thouraya Nabli Ajmi, Iheb Bougmiza, Ali Mtiraoui

**Affiliations:** 1 Department of Community Medicine - Medical Faculty of Sousse (Tunisia)

**Keywords:** General practice, problems managed by general practitioners, International Classification of Primary Care, ICPC-2

## Abstract

**Background:**

Primary health care is one of the most important pillars of the Tunisian health care system. However, very little information is available regarding the specificities of general practice and the patterns of morbidity encountered.

**Methods:**

We conducted a descriptive study from June 2002 to May 2003 in 85 primary health centres in Sousse during 12 randomly selected weeks in order to describe the variability of the morbidity in all seasons; (3 weeks were randomly selected in each season). Each working day of selected weeks, a systematic sample of patients was identified in each health centre by taking every fifth registered patient. There were 16,271 consultations. The International Classification of Primary Care (ICPC-2) was used to code recorded data of the consultation.

**Results:**

There were 24,882 reasons for encounter, a total of 18,097 problems managed by general practitioners (GPs), and 40,190 interventions. There was a predominance of females (62%) and a relatively young population attending the primary health care settings as 50% was aged less than 25 years. According to ICPC-2 chapters, we found that respiratory diseases were the main problems managed in primary health care (43%), followed by digestive (10.1%), locomotive (8.9%), cardiovascular affections (8.7%) and skin diseases (8.4%). These five conditions alone constituted about 80% of the total cases. However, genital conditions for both males and females (1%) as well as psychological and social problems (0.85%) were rarely managed in primary care.

**Conclusion:**

The findings will be useful in helping to revise the educational curriculum of medical studies as required in general practice and to plan relevant vocational training for GPs. They will also be important for health policy makers in Tunisia.

## Background

The primary health care system in Tunisia consists of a public sector and a private sector, and both are almost equivalent as regards the number of physicians and consultants. Patients are free to choose the private physician or the public health centre they prefer. Primary health centres are distributed in the whole territory of the country and constitute an important national ambulatory service network. However, very little information is available on the specificities of general practice and the patterns of morbidity encountered. Because another study is underway to learn more about morbidity in private settings, we decided to first investigate the morbidity patterns in the public sector.

The Department of Community Medicine at the Medical Faculty of Sousse carried out, for the first time in Tunisia, this one-year survey in all localities of the province of Sousse, which counted 505,370 inhabitants in 2002. This survey aimed to collect valid, complete, and representative data on reasons for encounter, problems managed by the general practitioner (GP) at the consultation, and interventions in managing these problems in primary health care. However, in this article, we are only considering one aspect of this survey, namely the problems managed by the GP at the consultation in general practice.

Therefore, we considered that the use of the International Classification of Primary Care (ICPC-2) was the most relevant tool for this study. The ICPC-2 allows classification of patient’s reasons for encounter (RFE), problems managed as labelled by the GP, and the GPsR17; interventions in managing these problems [1].

The objectives of the study were to illustrate the patterns of morbidity in general practice and to identify the main problems managed by the GPs at the consultation. This study presents no ethical issues; it was conducted in the context of a research unit and with the agreement of the regional health authorities of the Ministry of Health.

## Methods

### The study design

The study design was descriptive and cross-sectional. It was carried out in 85 primary health centres of the province of Sousse (Tunisia) among the 92 existing centres during one year, from June 2002 to May 2003, covering the four seasons.

### Sampling and study population

The study was conducted during 12 randomly selected weeks in order to describe the variability of the morbidity in all seasons; (3 weeks were randomly selected in each season). Each working day of selected weeks, a systematic sample of patients was identified in each health centre by taking every fifth registered patient. There were 16271 consultations, which were described according to a Subjective, Objective, Assessment, Plan (SOAP).

### Research instrument

The data collection instrument was designed to ease the recording process and ensure data quality. A first part was designed to be completed by the nurse in order to record administrative information about the health centre, as well as information describing patient’s socio-demographic profile (e.g., sex, age, health insurance). The second part of the instrument was used to collect information resulting from the doctor-patient encounter: reason of encounter, problems managed if any, and any other interventions related to the process of care. The second part was recorded by GPs themselves. All GPs who are working in the health centres were previously trained to conduct this research.

### Coding, data entry and analysis

The International Classification of Primary Care (ICPC-2) was used to code the data according to reasons for encounter, problems managed by the GP, and care related interventions at each consultation. Coding and data computer entry were performed by trained physicians in the department of community medicine. We used Epi-info (EPI 6) for data analysis and the significance level considered to determine whether or not the null hypothesis is rejected was 5%.

## Results

A total of 85 out of 92 primary health care centres in the region of Sousse were included as settings for data collection (in 7 health centres, physicians refused to take part of this survey for personal reasons).

### Characteristics of the study population

**Figure 1:**
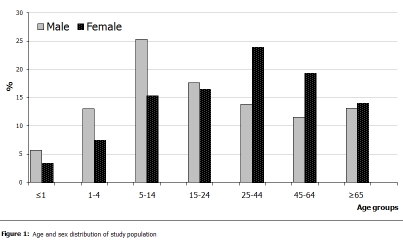
Age and sex distribution of study population

The population requiring primary health care in the public sector was predominantly female (62%). This proportion is significantly higher than in the general population (P<0.05), where the ratio female to male is 1.02. We found that 74% of patients were living in urban areas, a similar proportion to that of the general population (80%) (P= 0.31). The study population was relatively young with a mean age of 31.1 ± 24.3 years (the mean age in general population is 28.2 years). Children aged less than 15 years accounted for 33.1% (of whom 13.3% were aged less than 1 year), and those aged 65 years and over accounted for 13.7% of the surveyed consultations ( Figure 1 ).

The age-sex distribution of the study population showed that among young people aged less than 25 years, patients were predominantly male. In contrast, patients aged 25–64 years were predominantly female and the sex distribution of those aged 75 years and over was equally distributed ( Figure 1 ).

### Specificities of primary health care practice

#### Characteristics of consultations in general practice

A total of 81% of the problems managed were for a new health condition (first episode of care), 13.5% were for control of a chronic health condition such as hypertension and diabetes, and only 5.5% of patients were recalled for monitoring an acute illness managed by the GP at a previous consultation. The GPs classified 18% of the consultations as emergency cases.

#### Reasons for encounter, problems managed, and procedures (interventions) in general practice

There were 24,882 reasons for encounter (RFE) (1.9 ± 0.8 per encounter), with 18,097 health issues managed as diagnoses (1.3 ± 0.5 per encounter) and 40,190 interventions (procedures) (3.2 ± 1.9 per encounter). One RFE was given by the patient in 55.4% of cases; one single problem was managed in 82.6% of consultations, while at least two procedures or interventions were undertaken at 67.4% of consultations. In 63% of cases, the GPs decided that no monitoring or control visit was needed for the current episode of care. However, in 31% of cases, patients were asked to come back for a health control visit and only 5.5% were referred to a specialist.

#### Duration of encounters

The average consultation time was 6.8 minutes (± 3.8), and the mode = median = 5 minutes.

### Patterns of health problems managed in primary care

#### Health problems classified by ICPC Chapters

When health problems as managed by the GPs are listed in the order of frequency of the organ systems according to ICPC chapters, we found that respiratory diseases were the main problem managed in general practice (43%) followed by digestive (10.1%), locomotive (8.9%), cardiovascular affections (8.7%), and skin diseases (8.4%). These five affections alone constituted almost 80% of the total. However, genital affections for both males and females (1%), as well as psychological and social issues (0.85%), were rarely identified in primary care ( Table 1 ).

#### Health problems classified by ICPC rubrics

In addition to acute illnesses (tonsillitis acute: 15.7%, bronchitis acute: 11.2%, upper respiratory infections: 9.2%, and influenza: 3.5%), the two most frequent chronic diseases (high blood pressure and diabetes mellitus) were found within the top six, respectively in a proportion of 6.2% and 3.3%. However, asthma, which seemed to be a common chronic affection in Tunisia, was only managed in a proportion of 0.6% in general practice ( Table 2 ). The non-defined rubrics, as have been classified “others” by ICPC, represented 10.2% of all health problems managed in primary care.

#### The Top five health managed problems by ICPC chapters

We have taken into consideration the top eight chapters that included about 90% of all health problems which are managed by the GPs. Additional material, shows the most frequent managed problems (Top 5) in each of these chapters.

#### Distribution of health problems by sex

Respiratory diseases came in first place for both genders. However, skin diseases, which are ranked second, were more prevalent in males and digestive problems in females. The genital affections and psychological problems were at the bottom of the list, making them the less identified affections in primary health care.

#### Distribution of health problems by age group

The distribution of problems managed (additional material) was variable with age. Up to 24 years, the top three identified problems were respiratory affections, such as tonsillitis acute, upper respiratory infection, and acute bronchitis. Among children, under 5 years of age, the frequency of respiratory diseases was more than 60%, whereas digestive affections, such as diarrhoea, gastrointestinal infections, worms and parasites, came next and constituted 14%. For adults 25–44 years of age, respiratory affections remained the most frequent problem (34%), but high blood pressure was among the top 10 (1.6%) in ninth position.

For the age group of 45–64 years, uncomplicated hypertension was the top health problem (14.2%), followed by diabetes (9.3%) and acute bronchitis (8.5%). In this age group, locomotive (musculoskeletal) affections became frequent and constituted the fourth group of managed morbidity (10.2%).

Elderly population aged 65 years and over was characterized by an increase of chronic affections. High blood pressure was the most common disease (22.4%) and diabetes mellitus came in third place (7.9%). In addition, respiratory affections and locomotive illnesses were also frequent and presented, together, almost 30%.

## Discussion

### Strengths and limitations of the study

This is the first time such a wide morbidity study had been conducted in Tunisia. The results enabled us to identify the main health problems managed in primary health care in the province of Sousse. However, these results were mainly representative of morbidity at the public health sector because no private health settings have been included in this study. We also did not take into account the prevention activities, such as immunisation, ante-natal care and family planning, which are mainly being managed by nurses and midwives in these health centres. In addition, some health problems could not be classified and were thus coded as “other.” This was mainly noted in skin, digestive, and locomotive affections.

### Characteristics of general practice attendees

Sex distribution showed that 62% of patients were females. The predominance of the female population was reported in other developed and developing countries [2–5]. Female predominance, which is universal, is significant and could be explained by the fact that women are more anxious of their health, or maybe more vulnerable because of their reproductive health life (e.g., child bearing, genital activities, post partum). In addition, they are probably more available than men to attend the health centres or more able to manage the long waiting time at the health settings [2, 3].

Age distribution showed that one third of people attending general primary care consultations were children under 15 years and that half was aged under 25 years, which is relatively similar to the age distribution in the general population (49.2%). The elderly population aged 60 years and over constituted 17.6% of the total, which is significantly higher than in the general population (8.9%, P<0.05). Similar findings were reported in other countries [2]. The predominance of male children, especially, under five was noted in many other studies, particularly in countries that have similar cultures to that of Tunisia, where boys are still more welcome than girls [6, 7].

### Medical consultation

It was found that the great majority of consultations were for the first episode of a health problem (81%) rather than for control or monitoring of existing conditions (5.5%), which indicated that the demand for care, in general practice, was mainly for acute illnesses as reported in other studies [5, 6].

The average consultation time was 6.8 minutes (± 3.8) (mode = 5 minutes), and the duration varied from 1 to 30 minutes. Many other studies were interested in the length of consultation. In Estonia [9] for example, the average consultation lasted 9 minutes (± 4.9) and varied according to the number and the nature of problems, as well as with patient’s age, but was not influenced by gender. According to the same study, physical examination lasted about 2 minutes (± 1.9) and consultation time was longer for older age groups and for patients with psychological problems. In France, most of consultations in general practice lasted between 10 and 20 minutes, and 92% lasted less than 25 minutes (15 minutes: 37%, 10 minutes: 23%, 20 minutes: 19%). It has been also shown that the duration increased for chronic diseases, as well as for elderly people, and with female doctors [9].

### Patterns of problems managed in general practice

#### The most common problems managed

Health problems in the region of Sousse classified as “diagnoses” according to ICPC chapters showed that respiratory diseases were the most prevalent problem (43%). This finding is universally common as similar findings have been reported in many other studies [2, 4–5, 10–12], except in China, where high blood pressure and atherosclerosis ranked first [4]. The high proportion of respiratory diseases that have been noted in our study should be taken seriously in order to find out the main reasons of this predominance.

Some questions should be asked, such as, how are respiratory affections being taken care of in general practice, and how do GPs manage the use of antibiotics in the treatment of respiratory diseases? Theses questions are important because GPs tend to prescribe antibiotics for respiratory affections based on clinical arguments or merely to satisfy patients’ demand for antibiotic prescriptions [13]. As reported by Belgith [26], antibiotics were prescribed by GPs in the region of Monastir (Tunisia) to 50.4% of consulting patients of whom 52.8% did not require laboratory tests. The number of prescribed medicines as well as the non-respect of the therapeutic instructions constituted a potentially dangerous practice. This should justify the development of specific guidelines and training curricula for GPs, as well as vocational training courses that should be regularly assessed and updated [11]. Because tobacco use is high among young people and adults in Tunisia (20–40%), GPs should focus on educating people about the harmful effects of tobacco addiction and passive smoking on health.

A study conducted, in Souses [14], on physicians’ attitude towards tobacco use and prevention, concluded that their interest in health education regarding the harmful effects of tobacco depended on whether tobacco was involved in the current patient’s episode of care, and only 17.2% of physicians spontaneously provided advice to their patients on smoking cessation.

#### The most uncommon problems managed

Male and female genital affections accounted for only 1% of visits. Similar results were found in many other studies [2, 4, 12]. This low percentage could be explained by the presence of a midwife in each health centre, indicating that women may prefer them to male GPs. This issue also could be explained by the fact that genital complaints, in our culture, were often shyly declared and the GPs did not make the necessary effort to look beyond the declared symptom in order to unveil the hidden main reason for the encounter [15]. It was reported in other studies that this issue was related to a lack of doctor-patient communication or to an overcharge of medical consultations, hindering the quality of patient’s monitoring in primary health centres [3, 17].

It was also noted that social and psychological problems did not frequently emerge either as reason for encounter or as identified problem by the GPs (0.85%). This finding has also been noted in many other studies [2, 4, 12, 15]. Nandani de Silva [2] found that psychological problems accounted for only 1.9% in Sri Lanka, which was similar to findings in a Hong Kong survey [16]. According to an Australian survey, psychological illnesses accounted for 5.9% of diagnosed problems [16]. Surveys on psychiatric morbidity worldwide have shown that psychological problems, which accounted for about a third of all general practice consultation [2], were often missed by GPs. It was shown that GPs’ failure to detect psychological illnesses resulted because patients, more often, somatise their illness and express non specific complaints, which would be classified under other organic ICPC chapters and rubrics [2]. We should also note that the GPs were lacking specific training on mental health issues, so they rely on specialists to take care of patients presenting such illnesses. This argument would be confirmed as we know that Tunisian physicians have been trained according to a biomedical model, which had largely disregarded the psychological and social aspects of the health problem. It has been clearly shown that this issue constitutes a deep lack in general practice and that GPs need to enhance their knowledge and skills in this field [17, 18].

#### Health problems by age group

Health problems managed in general practice were variable with age [2, 19]. Children under five years of age, as shown in many other studies, were mainly suffering from respiratory diseases, such as upper respiratory tract infections, acute bronchitis, and tonsillitis acute [6, 7]. However, we should note that cardiovascular diseases (CVD), such as high blood pressure and diabetes mellitus started to appear early among young adults. In the age group of 25–44, these two chronic diseases were present, at the rates of 1.6% and 2.1%, respectively. In the age group of 45–64 years, uncomplicated hypertension came first (14.2%), followed by diabetes (9.3%). For elderly people aged 65 years and over, the occurrence of CVD became almost similar to the one in developed countries [4, 8, 19, 20]. This finding showed that the Tunisian population has been experiencing an epidemiological transition, where CVD are starting early and increasing in the general population. According to a recent study which aimed to assess CVD risk factors in urban schoolchildren in Sousse (Tunisia) [21], school children were exposed early to CVD risk factors, such as obesity (7.9%) and smoking (7.6%). It was also noted that among young people, high blood pressure and lipid disorders increased significantly with the weight. Therefore, the health system should be aware of the current and long term consequences of these diseases, such as degenerative complications and the increase of cardiovascular morbidity and mortality. Health managers should plan to set up preventive programmes and procedures for early diagnosis of cardiovascular affections [22], and GPs should be able to ensure health promotional activities, particularly for young people, to make them aware of the danger of cardiovascular risk factors related to people’ attitudes and behaviours, such as fast-food consumption, lack of physical exercise, and tobacco use [22, 23]. Therefore, reducing the prevalence and the incidence of cardiovascular morbidity and mortality should be done through an effective involvement of GPs in relevant community projects especially designed for preventive and promotional care [24].

## Conclusion

Primary health has very specific patterns that are different from what medical students are currently facing in hospitals during their vocational training. GPs in primary health care deal with a large scope of illnesses, including chronic diseases as well as preventive and promotional care. Therefore, we should engage a serious reflection on what the necessary scientific core curriculum should be for GPs and how this can help to improve the skills of our future GPs. [25, 27–29]. The findings will also interest health policy makers in Tunisia and regional primary health programme makers.

## Figures and Tables

**Table 1:** Diagnosed morbidity by ICPC Chapter

	** ICPC Chapters **	*** Frequency ***	** % **
** R **	Respiratory	** 7788 **	** 43.0 **
** D **	Digestive	1833	10.1
** L **	Locomotive (musculoskeletal)	1611	8.9
** K **	Cardiovascular	1574	8.7
** S **	Skin	1515	8.4
** T **	Endocrine and Metabolic	746	4.1
** F **	Eye	631	3.5
** A **	General	547	3.0
** U **	Urological	479	2.6
** H **	Ear	367	2.0
** B **	Blood	356	2.0
** N **	Neurological	312	1.7
** X **	Female genital	141	0.8
** P **	Psychological	136	0.8
** W **	Pregnancy, child-bearing and FP	31	0.2
** Y **	Male genital	22	0.1
** Z **	Social problems	8	0.0
	** Total **	** 18097 **	** 100.0 **

**Table 2:** The Top 20 diagnoses according to ICPC Rubrics

**ICPC *Code***	** ICPC Rubric name **	** Diagnosed Morbidity **		
		*** Frequency ***	*** % ***	*** Rank ***
** R76 **	Tonsillitis acute	2844	15.7	1
** R78 **	Acute bronchitis/bronchiolites	2032	11.2	2
** R74 **	Upper respiratory infection acute	1659	9.2	3
** K86 **	Hypertension uncomplicated	1124	6.2	4
** R80 **	Influenza	638	3.5	5
** T90 **	diabetes mellitus	589	3.3	6
** D70 **	Gastrointestinal infection	324	1.8	7
** U71 **	Cystitis/Urinary infection other	285	1.6	8
** F70 **	Conjunctivitis infectious	276	1.5	9
** L90 **	Osteoarthritis of knee	272	1.5	10
** S76 **	Skin infection other	252	1.4	11
** B82 **	Anaemia other/unspecified	232	1.3	12
** L20 **	Joint symptom/complaint NOS	221	1.2	13
** H70 **	Otitis externa	220	1.2	14
** A97 **	No disease	218	1.2	15
** S11 **	Skin infection post traumatic	208	1.1	16
** D22 **	Worms / Parasites	195	1.1	17
** D08 **	Flatulence/gas/belching	193	1.1	18
** L86 **	Back syndrome with radiating pain	188	1.0	19
** L91 **	Osteoarthritis others	163	0.9	20
** All other diagnoses **		5965	33.0	
*** Total ***		** 18075 **	** 100.0 **	

## Appendix

**Table 3:** The Top 5 diagnosis by ICPC chapters

** Respiratory Chapter **			
** Rank **	** ICPC Code **	** Diagnoses **	** % **
** 1 **	** R76 **	Tonsillitis acute	36.5
** 2 **	** R78 **	Acute bronchitis/bronchiolites	26.1
** 3 **	** R74 **	Upper respiratory infection acute	21.3
** 4 **	** R80 **	Influenza	8.2
** 5 **	** R75 **	Sinusitis acute/chronic	1.5
** Digestive Chapter **			
** Rank **	** ICPC Code **	** Diagnoses **	** % **
** 1 **	** D70 **	Gastrointestinal infection	17.7
** 2 **	** D22 **	Worms/Parasites	10.6
** 3 **	** D08 **	Flatulence/gas/belching	10.5
** 4 **	** D87 **	Stomach function disorder	8.5
** 5 **	** D82 **	Teeth/gum disease	7.5
** Locomotive (Musculoskeletal) Chapter **			
** Rank **	** ICPC Code **	** Diagnoses **	** % **
** 1 **	** L90 **	Osteoarthritis of knee	16.9
** 2 **	** L20 **	Joint symptom/complaint NOS	13.7
** 3 **	** L86 **	Back syndrome with radiating pain	11.7
** 4 **	** L91 **	Osteoarthritis others	10.1
** 5 **	** L99 **	Musculoskeletal disease, other	6.5
** Cardiovascular Chapter **			
** Rank **	** ICPC Code **	** Diagnoses **	** % **
** 1 **	** K86 **	Hypertension uncomplicated	71.4
** 2 **	** K88 **	Postural hypotension	7.6
** 3 **	** K71 **	Rheumatic fever/heart disease	4.3
** 4 **	** K96 **	Haemorrhoids	3.7
** 5 **	** K85 **	Elevated blood pressure	1.9
** Skin Chapter **			
** Rank **	** ICPC Code **	** Diagnoses **	** % **
** 1 **	** S76 **	Skin infection other	16.6
** 2 **	** S11 **	Skin infection post traumatic	13.7
** 3 **	** S98 **	Urticaria	6.9
** 4 **	** S72 **	Scabies/other acariasis	6.8
** 5 **	** S97 **	Chronic ulcer skin	5.9
** Endocrine and Metabolic Chapter **			
** Rank **	** ICPC Code **	** Diagnoses **	** % **
** 1 **	** T90 **	Diabetes mellitus	79.0
** 2 **	** T93 **	Lipid disorder	5.5
** 3 **	** T60 **	Results tests/procedures	2.9
** 4 **	** T86 **	Hypothyroidism/myxoedema	1.9
** 5 **	** T87 **	Hypoglycaemia	1.7
** Eye Chapter **			
** Rank **	** ICPC Code **	** Diagnoses **	** % **
** 1 **	** F70 **	Conjunctivitis infectious	43.7
** 2 **	** F71 **	Conjunctivitis allergic	16.2
** 3 **	** F91 **	Refractive error	10.3
** 4 **	** F72 **	Blepharitis/styp/chalazion	9.0
** 5 **	** F92 **	Cataract	5.7
** General Chapter **			
** Rank **	** ICPC Code **	** Diagnoses **	** % **
** 1 **	** A97 **	No disease	39.9
** 2 **	** A62 **	Administrative procedure	10.8
** 3 **	** A12 **	Allergy/allergic reaction	9.0
** 4 **	** A04 **	Weakness/tiredness general	6.2
** 5 **	** A72 **	Chickenpox	5.1

**Table 4:** The Top 5 diagnosis by age group

** < 1 year **				
** Rank **	** ICPC code **	** Diagnoses **	** Frequency **	** % **
** 1 **	** R74 **	Upper respiratory infection acute	236	31.8
** 2 **	** R78 **	Acute bronchitis/bronchiolites	154	20.8
** 3 **	** R76 **	Tonsillitis acute	26	3.5
** 4 **	** D70 **	Gastrointestinal infection	25	3.4
** 5 **	** H70 **	Otitis externa	21	2.8
** 1 – 4 years **				
** Rank **	** ICPC code **	** Diagnoses **	** Frequency **	** % **
** 1 **	** R74 **	Upper respiratory infection acute	477	27.9
** 2 **	** R78 **	Acute bronchitis/bronchiolites	354	20.7
** 3 **	** R76 **	Tonsillitis acute	246	14.4
** 4 **	** D70 **	Gastrointestinal infection	60	3.5
** 5 **	** D22 **	Worms/Parasites	36	2.1
** 5 – 14 years **				
** Rank **	** ICPC code **	** Diagnoses **	** Frequency **	** % **
** 1 **	** R76 **	Tonsillitis acute	1075	32.5
** 2 **	** R74 **	Upper respiratory infection acute	441	13.3
** 3 **	** R78 **	Acute bronchitis/bronchiolites	433	13.1
** 4 **	** D22 **	Worms/Parasites	119	3.6
** 5 **	** R80 **	Influenza	93	2.8
** 15 – 24 years **				
** Rank **	** ICPC code **	** Diagnoses **	** Frequency **	** % **
** 1 **	** R76 **	Tonsillitis acute	607	20.8
** 2 **	** R78 **	Acute bronchitis/bronchiolites	266	9.1
** 3 **	** R74 **	Upper respiratory infection acute	193	6.6
** 4 **	** R80 **	Influenza	158	5.4
** 5 **	** D70 **	Gastrointestinal infection	82	2.8
** 25 – 44 years **				
** Rank **	** ICPC code **	** Diagnoses **	** Frequency **	** % **
** 1 **	** R76 **	Tonsillitis acute	535	15.3
** 2 **	** R78 **	Acute bronchitis/bronchiolites	299	8.6
** 3 **	** R80 **	Influenza	180	5.2
** 4 **	** R74 **	Upper respiratory infection acute	166	4.8
** 5 **	** B82 **	Anaemia other/unspecified	97	2.8
** 6 **	** T90 **	Diabetes mellitus	74	2.1
** 45 – 64 years **				
** Rank **	** ICPC code **	** Diagnoses **	** Frequency **	** % **
** 1 **	** K86 **	Hypertension uncomplicated	428	14.2
** 2 **	** T90 **	Diabetes mellitus	281	9.3
** 3 **	** R78 **	Acute bronchitis/bronchiolites	258	8.5
** 4 **	** R76 **	Tonsillitis acute	226	7.5
** 5 **	** L90 **	Osteoarthritis of knee	114	3.8
** 65 years and over **				
** Rank **	** ICPC code **	** Diagnoses **	** Frequency **	** % **
** 1 **	** K86 **	Hypertension uncomplicated	604	22.4
** 2 **	** R78 **	Acute bronchitis/bronchiolites	247	9.2
** 3 **	** T90 **	Diabetes mellitus	212	7.9
** 4 **	** L90 **	Osteoarthritis of knee	127	4.7
** 5 **	** R76 **	Tonsillitis acute	110	4.1
